# P-1269. Non-Vaccine Group B *Streptococcus* Serotypes in Clinical Samples

**DOI:** 10.1093/ofid/ofae631.1450

**Published:** 2025-01-29

**Authors:** Allison A Taffet, Megan Job, Sydney Haldeman, Hervé Tettelin, Adam J Ratner

**Affiliations:** New York University Grossman School of Medicine, New York, New York; New York University Grossman School of Medicine, New York, New York; New York University Grossman School of Medicine, New York, New York; Institute for Genome Sciences, University of Maryland School of Medicine, Baltimore, Maryland; New York University School of Medicine, New York, NY

## Abstract

**Background:**

Group B *Streptococcus* (GBS) is a common colonizer of the gastrointestinal and genitourinary tracts and a major cause of neonatal infection. Currently, pregnant individuals are screened for GBS colonization in late pregnancy and given intrapartum antibiotics if positive, but this approach is resource-intensive and incompletely effective. GBS has also emerged as a pathogen in non-pregnant adults. A hexavalent vaccine candidate (GBS6) that protects against 6 of the 10 known GBS serotypes (vaccine-type [VT] serotypes: Ia, Ib, II-V; non-vaccine type [NVT] serotypes: VI-IX) is in phase II/III trials. However, emergence of NVT serotypes could threaten the overall impact of GBS6. Previous studies have estimated that < 2% of GBS isolates are NVT.

**Figure:**
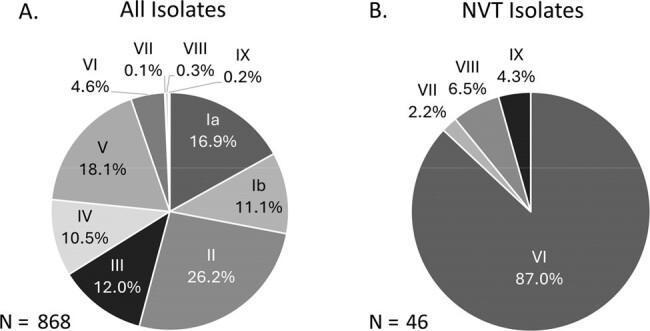

A. Serotype distribution for all isolates, from both colonization and sterile sites. B. Distribution of NVT isolates.

**Methods:**

We conducted laboratory-based surveillance for GBS isolates from sterile sites and rectovaginal colonization samples from May 2021 – December 2023. Urinary samples were not included. GBS whole genome sequencing was used to determine serotype, multilocus sequence type (ST), and clonal complex (CC). We performed retrospective chart review to gather demographic and clinical data.

**Results:**

For this interim analysis, 873 isolates were successfully sequenced, of which 868 had a detectable serotype (Figure). 5.3% of isolates were NVT. NVT isolates were present among both colonization (6.8%) and sterile-site isolates (4.1%). The majority of NVT isolates (40/46; 87%) were serotype VI. Serotypes VII – IX were rarely observed. Of the serotype VI isolates, almost all (37/40; 92.5%) were CC1. 34 serotype VI isolates had antibiotic susceptibility data available from the microbiology lab. 79% of these were susceptible to tetracycline, compared to the all-serotype average of 18%. 79% were susceptible to clindamycin, higher than the all-serotype average of 49%. All isolates from neonatal invasive disease (N=15) were VT.

**Conclusion:**

The majority of clinical colonization and sterile-site GBS isolates have serotypes contained in GBS6. However, NVT rates are higher than previously estimated, primarily driven by serotype VI. Serotype VI merits further study and consideration for addition to future GBS vaccine candidates.

**Disclosures:**

**Hervé Tettelin, PhD**, Merck, Sharpe & Dohme: Advisor/Consultant

